# Asymmetric Side-Group Engineering of Nonfused Ring Electron Acceptors for High-Efficiency Thick-Film Organic Solar Cells

**DOI:** 10.1007/s40820-025-01905-y

**Published:** 2025-11-10

**Authors:** Dawei Li, Nan Wei, Ya-Nan Chen, Xiaodong Wang, Xu Han, Ziqing Bian, Xinyuan Zhang, Zhe Zhang, Wenkai Zhang, Xinjun Xu, Cuihong Li, Yahui Liu, Hao Lu, Zhishan Bo

**Affiliations:** 1 Key Laboratory of Energy Conversion and Storage Materials, College of Chemistry, Beijing Normal University, Beijing, 100875 People’s Republic of China; 2 College of Textiles and Clothing, State Key Laboratory of Bio-Fibers and Eco-Textiles, Qingdao University, Qingdao, 266071 People’s Republic of China; 3 College of Materials Science and Engineering, Qingdao University, Qingdao, 266071 People’s Republic of China; 4 Department of Physics and Applied Optics Beijing Area Major Laboratory, Beijing Normal University, Beijing, 100875 People’s Republic of China; 5 Laboratory of Eco-environmental Polymer Materials of Gansu Province, College of Chemistry and Chemical Engineering, Northwest Normal University, Lanzhou, 730070 People’s Republic of China

**Keywords:** Organic solar cells, Nonfused ring electron acceptors, Asymmetric, Power conversion efficiency

## Abstract

A nonfused ring electron acceptor (NFREA), designated as **TT-Ph-C6**, has been synthesized with the aim of enhancing the power conversion efficiency (PCE) of organic solar cells (OSCs). By integrating asymmetric phenylalkylamino side groups, **TT-Ph-C6** demonstrates excellent solubility and its crystal structure exhibits compact packing structures with a three-dimensional molecular stacking network. These structural attributes markedly promote exciton diffusion and charge carrier mobility, particularly advantageous for the fabrication of thick-film devices. **TT-Ph-C6**-based devices have attained a PCE of 18.01% at a film thickness of 100 nm, and even at a film thickness of 300 nm, the PCE remains at 14.64%, surpassing that of devices based on 2BTh-2F. These remarkable properties position **TT-Ph-C6** as a highly promising NFREA material for boosting the efficiency of OSCs.

**Supplementary Information:**

The online version contains supplementary material available at 10.1007/s40820-025-01905-y.

## Highlights

- The asymmetric side-group strategy was employed to develop a nonfused ring electron acceptor, designated as **TT-Ph-C6**, exhibiting enhanced solubility and three-dimensional molecular stacking.
- Strong *π*–*π* interactions optimized blend film morphology, enabling **TT-Ph-C6**-based devices to achieve a power conversion efficiency (PCE) of 18.01% and FF of 80.10%, surpassing the 16.78% PCE of symmetric-chain 2BTh-2F.
- Extended exciton diffusion lengths and accelerated dissociation further endowed **TT-Ph-C6** with exceptional thick-film tolerance, delivering 15.18% PCE at 200 nm and 14.64% at 300 nm—among the highest efficiencies reported for non-fused acceptors.

**Supplementary Information:**

The online version contains supplementary material available at 10.1007/s40820-025-01905-y.

## Graphical abstract

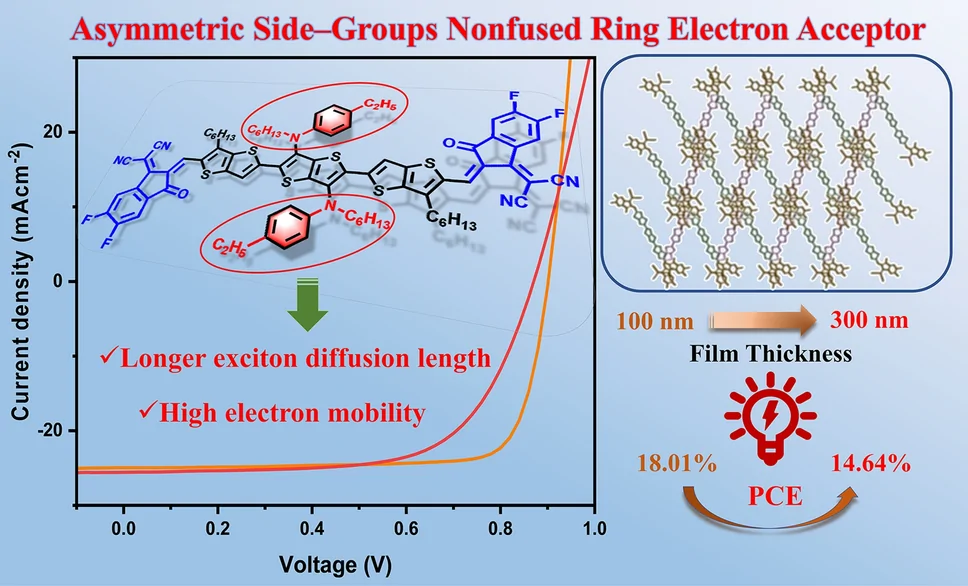

## Introduction

Organic solar cells (OSCs) have garnered significant interest due to their remarkable attributes, such as simple structures, exceptional flexibility, lightweight feature, and compatibility with roll-to-roll printing processes [1–10]. These unique characteristics highlight the vast potential of OSCs for portable electronic devices and versatile photovoltaic technologies. In recent times, fused ring electron acceptors (FREAs), particularly the Y-series, have made substantial progress, elevating the power conversion efficiency (PCE) of single-junction OSCs to nearly 20% [11–18]. However, the synthesis of FREAs often involves inefficient ring-closure reactions and requires complex, multi-step synthetic procedures, thereby increasing both the complexity of preparation and associated costs. Hence, NFREAs start to gain more and more attention. They circumvent ring-closure reactions and offer a straightforward synthesis process. Recent research findings indicate that NFREAs have made significant strides, achieving a PCE surpassing 19%, thus narrowing the performance gap with FREAs [19]. Nevertheless, several key challenges still need to be overcome to expedite the commercialization of NFREAs.

Acceptor–(donor–acceptor′–donor)–acceptor (A–D–A′–D–A)-type Y-series acceptors, representative of FREAs, exhibit a curved molecular configuration and a distinct stacking mode, forming a crystal structure network rich in charge transport channels and interconnected nodes. This structural feature facilitates efficient exciton and charge transport, substantially enhancing the exciton diffusion length and endowing thick-film OSCs with exceptional photovoltaic performance [20–28]. In contrast, while NFREAs possess a more streamlined molecular architecture, constructed through single-bond linkages resulting in acceptor–donor–acceptor (A–D–A) or more intricate A–D–A′–D–A structures, this structural attribute actually enables precise adjustment of the core structure and *π*-bridge units, allowing for meticulous control over photoelectric properties like light absorption, energy levels, and electron mobility. Moreover, in the design of NFREAs, intramolecular non-covalent interactions, such as S⋯O, S⋯S, and O⋯H, are frequently incorporated to effectively tackle challenges associated with the planarity and rigidity of the molecular framework, thereby promoting efficient molecular stacking [29–34]. However, it is worth noting that the electron mobility of NFREAs is generally lower than that of Y-series acceptors. Consequently, to further augment charge transport abilities, it is imperative to enhance molecular rigidity and promote closer packing. One effective strategy to achieve this is the introduction of asymmetric side groups. Asymmetric side groups can significantly influence the molecular packing and electronic properties of NFREAs. By carefully designing these side groups, it is possible to enhance the molecular rigidity and promote closer packing, which in turn improves charge transport and exciton diffusion [35–37]. However, the currently available high-performance non-fused ring electron acceptors typically incorporate large sterically hindered side groups, such as diphenylamine and 2,4,6-triisopropylbenzene moieties, which are effective in mitigating excessive aggregation and ensuring satisfactory solubility [38–43]. Nevertheless, these bulky side chains also hinder close molecular packing, leading to reduced crystallinity and packing density of the acceptor materials. Consequently, this poses challenges to charge transport and exciton diffusion. Given the necessity for acceptor materials in thick-film OSCs to exhibit both high charge carrier mobility and good solubility [44], it becomes crucial to finely tune the sterically hindering side groups during the design of non-fused ring acceptor molecules. This delicate balance between the two performance requirements is of utmost importance in the development of high-performance NFREAs, especially when constructing high-performance thick-film devices based on NFREAs, where the significance of this balancing act is particularly evident.

Herein, we report a novel NFREA, designated as **TT-Ph-C6**, which exhibits significantly enhanced solubility and promotes the formation of a compact, ordered three-dimensional (3D) network stacking structure through the innovative incorporation of asymmetric phenylalkylamine side groups. This structural feature effectively enhances charge transport and key device parameters, including short-circuit current density (*J*_sc_) and fill factor (FF). OSCs incorporating the **TT-Ph-C6** acceptor have attained a remarkable PCE of 18.01%, with an FF exceeding 80%, setting a new benchmark in the field of NFREAs. In comparison to the 2BTh-2F acceptor molecule featuring diphenylamine side groups, **TT-Ph-C6**-based devices exhibit a more gradual efficiency decline as active layer thickness varies. Specifically, at thicknesses of 100, 200, and 300 nm, PCEs are maintained at 18.01%, 15.18%, and 14.64%, respectively, highlighting its exceptional thickness tolerance. This underscores the crucial role of **TT-Ph-C6** in the fabrication of thick-film devices for NFREA-based OSCs.

## Experimental Section

### Materials Synthesis and Characterization

**Fig. 1:**
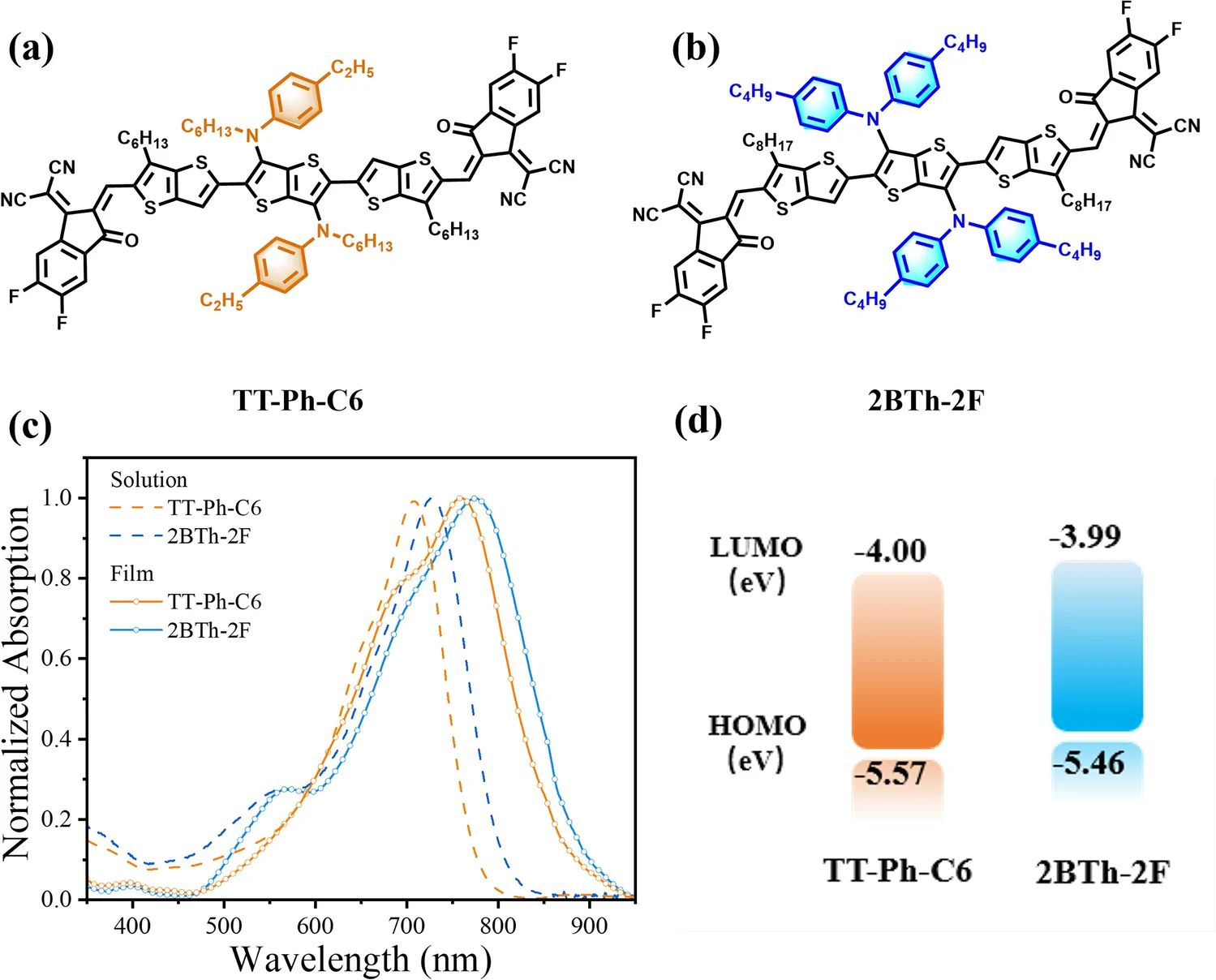
Chemical structure of **a TT-Ph-C6** and **b** 2BTh-2F; **c** UV–Vis absorption spectra (in chloroform solutions and as films) and **d** energy level diagram of **TT-Ph-C6** and 2BTh-2F

**Scheme 1:**
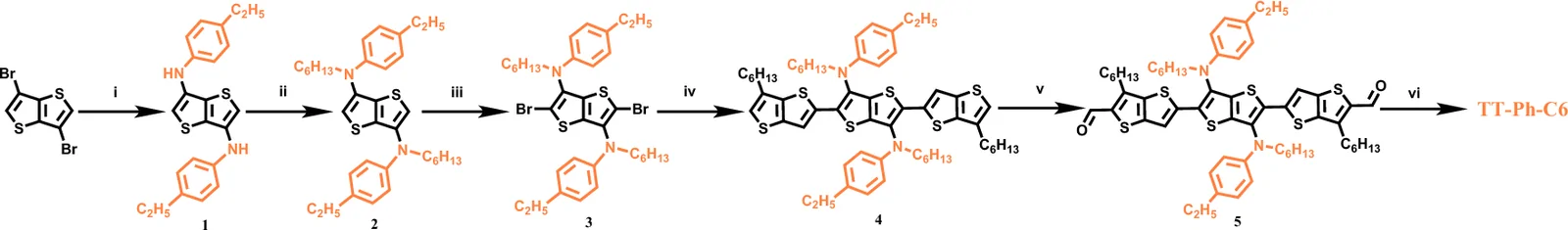
Chemical structures and synthetic routes of **TT-Ph-C6.** Reagents and conditions: (1) 4-ethylaniline, Pd(OAc)_2_, NaOt-Bu, X-phos, toluene, 110 °C; (2) 1-iodohexane, NaH, DMF; (3) NBS, DMF, 0 °C; (4) trimethyl(6-hexylthieno[3,2-*b*]thiophen-2-yl)stannane, Pd(PPh_3_)_4_, toluene, 110 °C; (5) POCl_3_, 1,2-dichloroethane, DMF, 80 °C; (6) 2-(5,6-difluoro-3-oxo-2,3-dihydro-1*H*-inden-1-ylidene)malononitrile, acetic anhydride, toluene

The molecular structure and the detailed synthetic route of the asymmetric NFREA **TT-Ph-C6** are shown in Fig. 1a and Scheme 1, respectively, whereas the chemical structure of the non-fused ring acceptor 2BTh-2F is illustrated in Fig. 1b. The detailed description of the synthetic procedure for **TT-Ph-C6** is provided in the Supporting Information (SI). The synthesis initiated with an efficient Buchwald–Hartwig coupling reaction between 3,6-dibromothiophene[3,2-*b*]thiophene and 4-ethyl-*N*-hexylaniline, yielding compound 1 with a high yield of 88%. Subsequently, compound 1 was facilely transformed into compound 2 via a Hofmann alkylation reaction with 1-iodohexane, achieving a remarkable yield of 95%. This was followed by a bromination step, which converted compound 2 into compound 3 with a yield of 90%. Next, we employed the Stille reaction to couple compound 3 with tributyl(6-hexylthiophene[3,2-*b*]thiophene-2-yl)stannane, resulting in the successful synthesis of compound 4 without purification. Compound 5 was then efficiently converted into compound 4 through the Vilsmeier–Haack reaction, attaining a yield of 60% for tow steps. Finally, compound 5 was subjected to a Knoevenagel condensation reaction with 3-(1,1-dicyanomethylene)-5,6-difluoro-1-indanone, leading to the successful synthesis of the target acceptor **TT-Ph-C6** with an impressive yield of 90%. **TT-Ph-C6** exhibits good solubility in commonly used organic solvents, and its chemical structure has been rigorously verified by ^1^H and ^13^C NMR spectroscopy, as well as mass spectrometry.

## Results and Discussion

### Photophysical and Electrochemical Properties

Figure 1c displays the normalized ultraviolet–visible (UV–vis) absorption spectra for the two acceptor molecules, **TT-Ph-C6** and 2BTh-2F. In their solution states, **TT-Ph-C6** and 2BTh-2F exhibit maximum absorption peaks at 707 and 726 nm, respectively. The stronger electron-donating ability of bis(4-butylphenyl)amino in 2BTh-2F, compared to alkylphenylamino in **TT-Ph-C6**, induces a more prominent intramolecular charge transfer (ICT) effect, leading to a red shift of the absorption peak of 2BTh-2F. When transitioning to the film state, the maximum absorption peaks of **TT-Ph-C6** and 2BTh-2F shift to 760 and 773 nm, respectively. Notably, a distinct shoulder peak is observed at approximately 690 nm in the **TT-Ph-C6** film absorption spectrum, suggesting a more advantageous molecular stacking for **TT-Ph-C6**. Utilizing the equation *E*_g_^opt^ = 1240/*λ*_onset_, we subsequently determined the optical bandgaps of **TT-Ph-C6** and 2BTh-2F to be 1.42 and 1.39 eV, respectively. Concurrently, electrochemical cyclic voltammetry (CV) was employed to assess the energy levels of the NFREAs (see Fig. S1 for details). As illustrated in Fig. 1d and Table 1, the highest occupied molecular orbital (HOMO) and lowest unoccupied molecular orbital (LUMO) energy levels for **TT-Ph-C6** and 2BTh-2F are − 5.57/4.00 and − 5.46/ − 3.99 eV, respectively.

**Table 1:** Optical and electronic properties of **TT-Ph-C6** and 2BTh-2F

Acceptor	*λ*_max_ (nm)^a^	*λ*_max_ (nm)^b^	*λ*_onset_ (nm)^b^	*E*_g_^opt^ (eV)^b^	HOMO (eV)	LUMO (eV)
**TT-Ph-C6**	707	760	868	1.43	− 5.57	− 4.00
2BTh-2F	726	773	890	1.39	− 5.46	− 3.99

^a^In dilute chloroform solution

^b^As thin film

### Single-Crystal Analysis Photovoltaic Properties and Stability

**Fig. 2:**
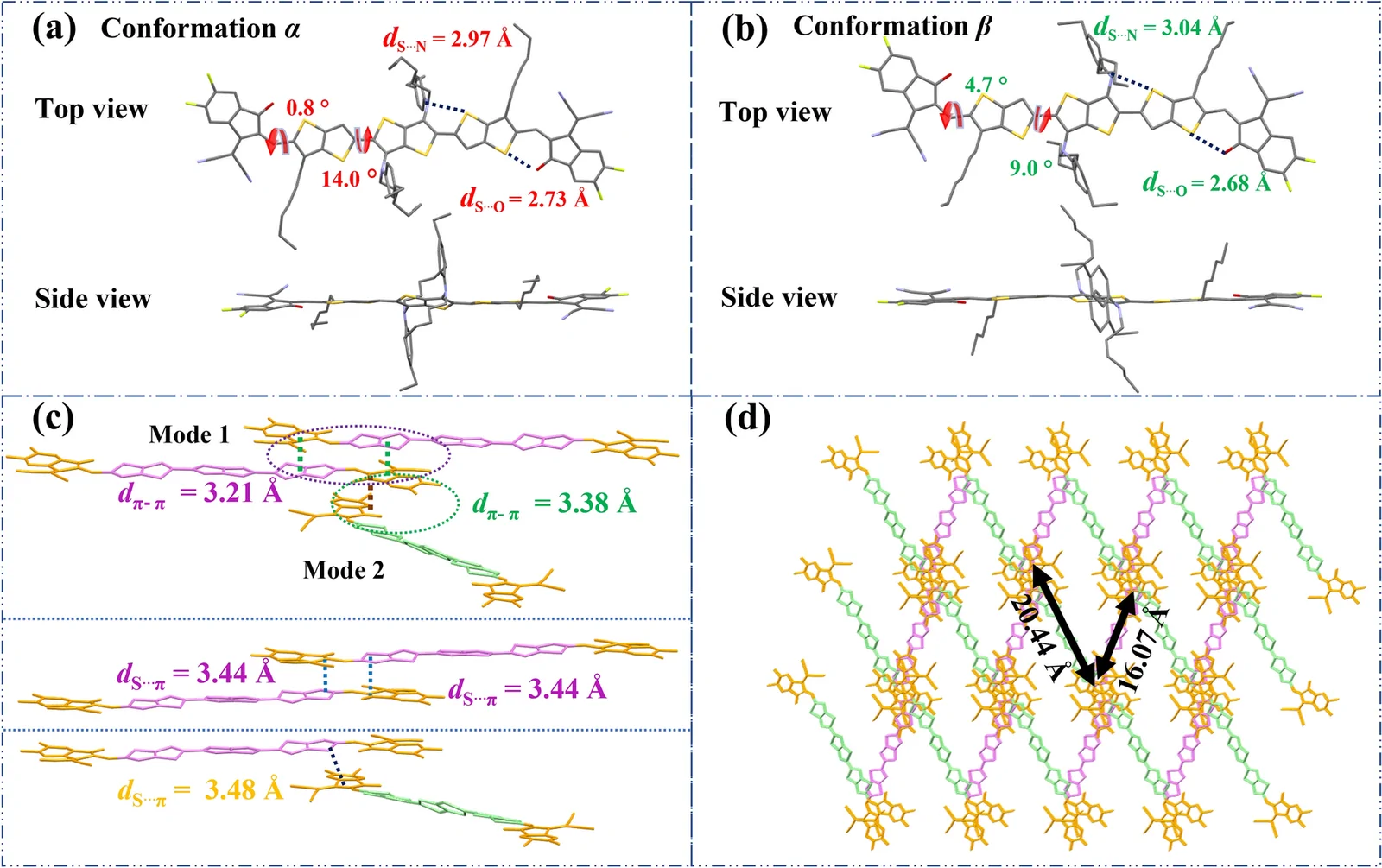
Single-crystal structures of **TT-Ph-C6** with two conformations (**a** conformation *α* and **b** conformation *β*), **c** molecular packing modes, **d** 3D stacking network in single crystals. Conformations are depicted in two colors: pink (conformation *α*) and green (conformation *β*). (Color figure online)

The crystal structure of acceptor molecules exerts a significant influence on the photovoltaic performance of OSCs. To delve deeply into the single-crystal structural characteristics of these acceptor molecules, we conducted X-ray diffraction measurements and analysis on the **TT-Ph-C6** single crystal. As depicted in Fig. 2a, b, the acceptor adopts two distinct conformations within the unit cell, designated as *α* and *β*. Notably, in both the *α* and *β* conformations, the torsion angles between the external thiophene[3,2-*b*]thiophene moieties and the terminal groups are remarkably small, measuring 0.8° and 4.7° respectively, thereby demonstrating the high structural stability. Upon further examination, it was revealed that the intramolecular S⋯O distances in the *α* and *β* conformations are 2.73 and 2.68 Å, respectively, which are considerably lower than the sum of the van der Waals radii for sulfur and oxygen (*r*_w,S_⋯_N_ = 3.25 Å) [32]. This provides compelling evidence for the presence of pronounced S⋯O = C interactions within the molecule. Moreover, we determined the S···N distances in the *α* and *β* conformations to be 2.97 and 3.04 Å, respectively, both of which are substantially below the sum of the van der Waals radii for sulfur and nitrogen (*r*_w,S_⋯_N_ = 3.50 Å), indicating the occurrence of S···N non-covalent interactions. The single-crystal structure of **TT-Ph-C6**, as depicted in Figs. 2c and S2, illustrates the coexistence of two conformations and provides insights into how asymmetric side chain structures influence molecular interactions. Within the crystal cell, two distinct *π*–*π* stacking modes are identified between adjacent molecules. Mode 1 features parallel-sliding *π*–*π* stacking between pairs of *α* conformations, while Mode 2 involves *π*–*π* stacking between one *α* and one *β* conformation. The respective *π*–*π* stacking distances (*d*_*π*–*π*_) for Modes 1 and 2 are 3.21 and 3.38 Å. Additionally, S⋯*π* interactions are observed, with distances of 3.44 Å between adjacent *α* conformations and 3.48 Å between *α* and *β* conformations. As illustrated in Fig. 2d, the crystal structure exhibits a 3D molecular stacking network interlaced with parallelogram-shaped voids measuring 20.44 × 16.07 Å^2^. This architecture confers stability and compactness to the material, facilitating the formation of a more efficient 3D electron transport network, which is crucial for enhancing electron mobility. The single-crystal structure analysis of 2BTh-2F, functionalized with bis(4-butylphenyl)amino side groups, reveals a characteristic interfacial torsion angle of 6.13° between its thiophene backbone segment and the terminal thiophene[3,2-*b*] heterocyclic unit, reflecting subtle conformational adjustments within the molecular architecture. Intramolecular stabilization is mediated by two distinct noncovalent interactions: an S⋯N contact (3.07 Å) and a notably shorter S⋯O interaction (2.78 Å), both contributing to structural rigidity. Furthermore, the crystalline packing arrangement features an interplanar *π*–*π* stacking distance of 3.39 Å between adjacent molecular planes [29]. Comparative structural analysis highlights that **TT-Ph-C6** demonstrates enhanced noncovalent synergy, with both intensified S⋯O/S⋯N interactions and an optimized molecular backbone planarity. Moreover, **TT-Ph-C6** has a smaller *π*–*π* stacking distance, indicative of a more compact *π*–*π* packing arrangement. These characteristics endow **TT-Ph-C6** with a more robust and efficient 3D electron transport network, which is conducive to enhancing charge transfer efficiency—a critical factor for achieving high *J*_sc_ and FF in OSCs (vide infra).

### Photovoltaic Properties

**Fig. 3:**
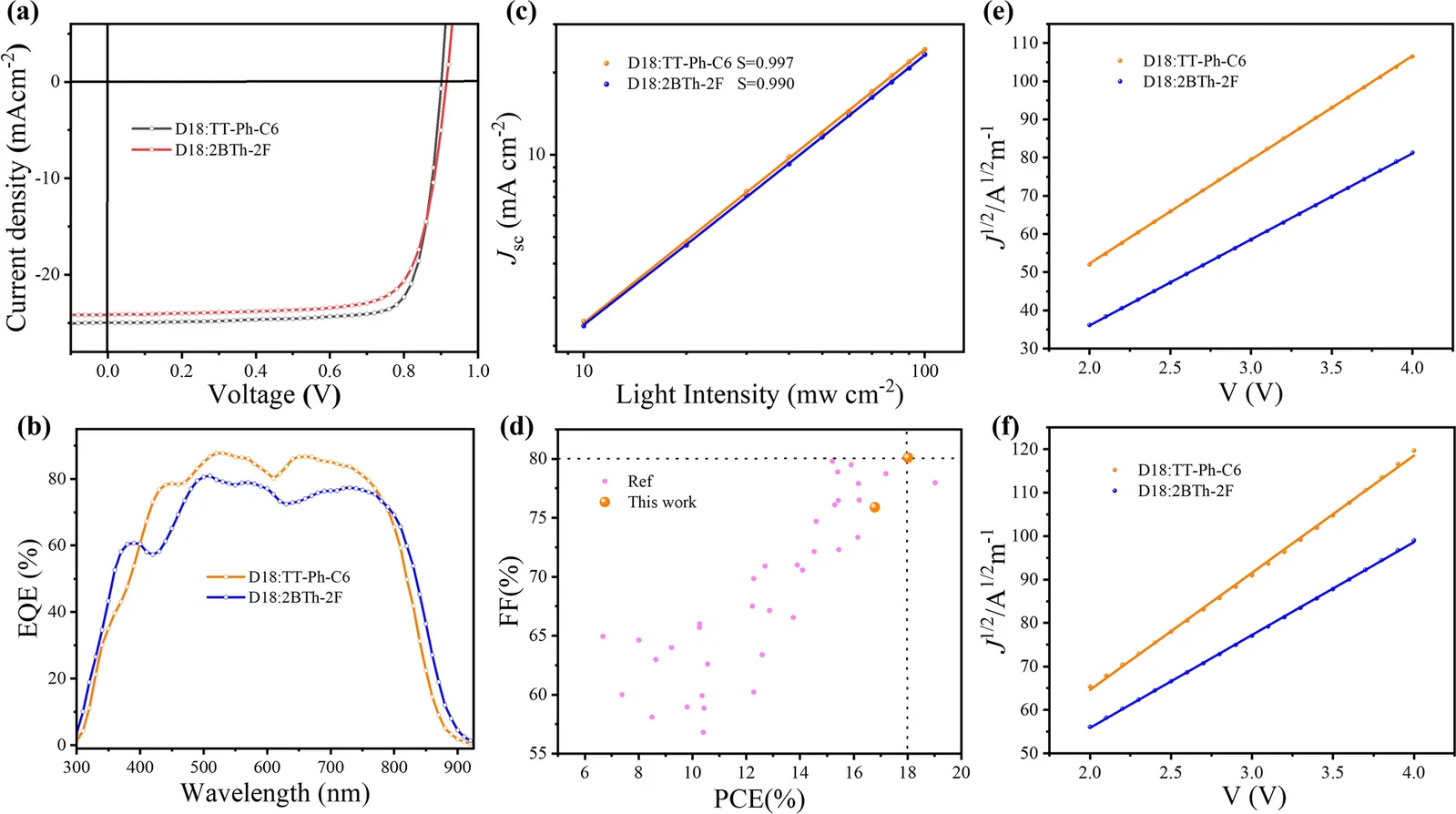
Performance and photoelectric characteristics of the **TT-Ph-C6** and 2BTh-2F-based devices, **a** *J*–*V* curves, **b** EQE spectra, **c** dependence of *J*_sc_ on *P*_light_, **d** recently reported photovoltaic performance statistics of OSCs based on NFREAs, **e** the *μ*_h_ transport mobilities; **f** *μ*_e_ transport mobilities

Conventional OSCs, configured with ITO/2PACz/polymer:acceptor/PDINN/Ag, were fabricated to assess the photovoltaic performances of **TT-Ph-C6** and 2BTh-2F. The classic polymer D18 was selected as the donor owing to its compatible energy level and complementary absorption with the acceptors. Detailed optimization steps for fabricating optimal devices, including the selection of the *D*/*A* ratio, annealing temperature, and additives, are provided in the supplementary information. The current density–voltage (*J*–*V*) characteristic curves for the optimized devices are depicted in Fig. 3a and summarized in Table 2. Our results indicate that **TT-Ph-C6**-based devices exhibit the highest PCE of 18.01%, with a *V*_oc_ of 0.90 V, a *J*_sc_ of 24.96 mA cm^−2^, and an FF of 80.10%. In contrast, 2BTh-2F-based devices deliver a lower PCE of 16.78%, with a *V*_oc_ of 0.91 V, a *J*_sc_ of 24.14 mA cm^−2^, and an FF of 75.90%. As shown in Fig. 3b, the external quantum efficiency (EQE) spectra of the devices based on these two acceptors are presented. It is evident that the higher *J*_sc_ value in **TT-Ph-C6**-based OSCs is primarily attributed to their higher EQE value (23.63 mA cm^−2^), which peaks at 87%. In comparison, 2BTh-2F-based devices exhibit the highest EQE value (23.18 mA cm^−2^) of 81%. Furthermore, the EQE spectrum of **TT-Ph-C6**-based OSCs reveals a broad response range from 300 to 860 nm, slightly narrower than that of the 2BTh-2F-based devices, which is consistent with their absorption spectrum.

**Table 2:** Photovoltaic parameters of devices based on **TT-Ph-C6** and 2BTh-2F

Active layer	*V*_OC_ (V)	*J*_SC_ (mAcm^−2^)	FF (%)	PCE (%)
D18:**TT-Ph-C6**	0.90	24.96	80.10	18.01 (17.75 ± 0.15)^a^
D18:2BTh-2F	0.91	24.14	75.90	16.78 (16.38 ± 0.31)^a^

^a^Average PCE of 10 device

Through an extensive investigation into the charge recombination mechanisms, we gain a deeper understanding of the photovoltaic properties of devices incorporating asymmetric NFEAs. By scrutinizing the relationship between *J*_sc_ and light intensity (*P*_light_), which follows the power law *J*_sc_ ∞ *P*_light_^*α*^, we can elucidate the charge recombination processes. Here, *α* denotes the bimolecular recombination index, ascertainable from the slope of the log–log plot of *J*_sc_ versus Plight [45–47]. As depicted in Fig. 3c, a dual logarithmic plot of *J*_sc_ against *V*_oc_ reveals that the *α* value for devices based on **TT-Ph-C6** attains 0.997. This value is notably higher than that of devices based on 2BTh-2F (0.990) and approaches the ideal unity, signifying well-suppressed bimolecular recombination within the **TT-Ph-C6**-based devices. Moreover, we carried out a detailed examination of the photocurrent density (*J*) versus effective voltage (*V*_eff)_ relationship in these devices, with the detailed findings presented in Fig. S3. The D18:**TT-Ph-C6** device exhibits an impressive exciton dissociation efficiency (*P*_diss_) of 96% and a charge collection efficiency (*P*_coll_) of 87%. These results underscore the superior performance of **TT-Ph-C6**-based devices in exciton dissociation and charge transport, effectively mitigating recombination, thereby yielding elevated *J*_sc_ and FF. Furthermore, we have compiled the latest research data on FF and PCE for devices employing NFREAs. In comparison, our study achieves a higher FF (as illustrated in Fig. 3d), marking the highest reported FF for NFREA-based OSCs thus far.

To investigate the charge transport characteristics of the asymmetric acceptor, the space-charge-limited current (SCLC) method was employed. The hole mobility (*μ*_h_) and electron mobility (*μ*_e_) were estimated using the standard device structures: ITO/PEDOT:PSS/active layer/Au for hole transport and ITO/ZnO/active layer/Al for electron transport, respectively. As shown in Fig. 3e, f, and Table S1, the *μ*_h_/*μ*_e_ values for D18:**TT-Ph-C6** and D18:2BTh-2F blend films are estimated to be 2.44 × 10^−4^/2.48 × 10^−4^ and 1.53 × 10^−4^/1.71 × 10^−4^ cm^2^ V^−1^ s^−1^, respectively, resulting in corresponding *μ*_h_/*μ*_e_ values of 0.98 and 0.89. The higher and more balanced hole and electron mobilities are the reasons for the improvement in *J*_sc_ and FF of the D18:**TT-Ph-C6** device.

### Charge Transfer and Exciton Lifetimes

**Fig. 4:**
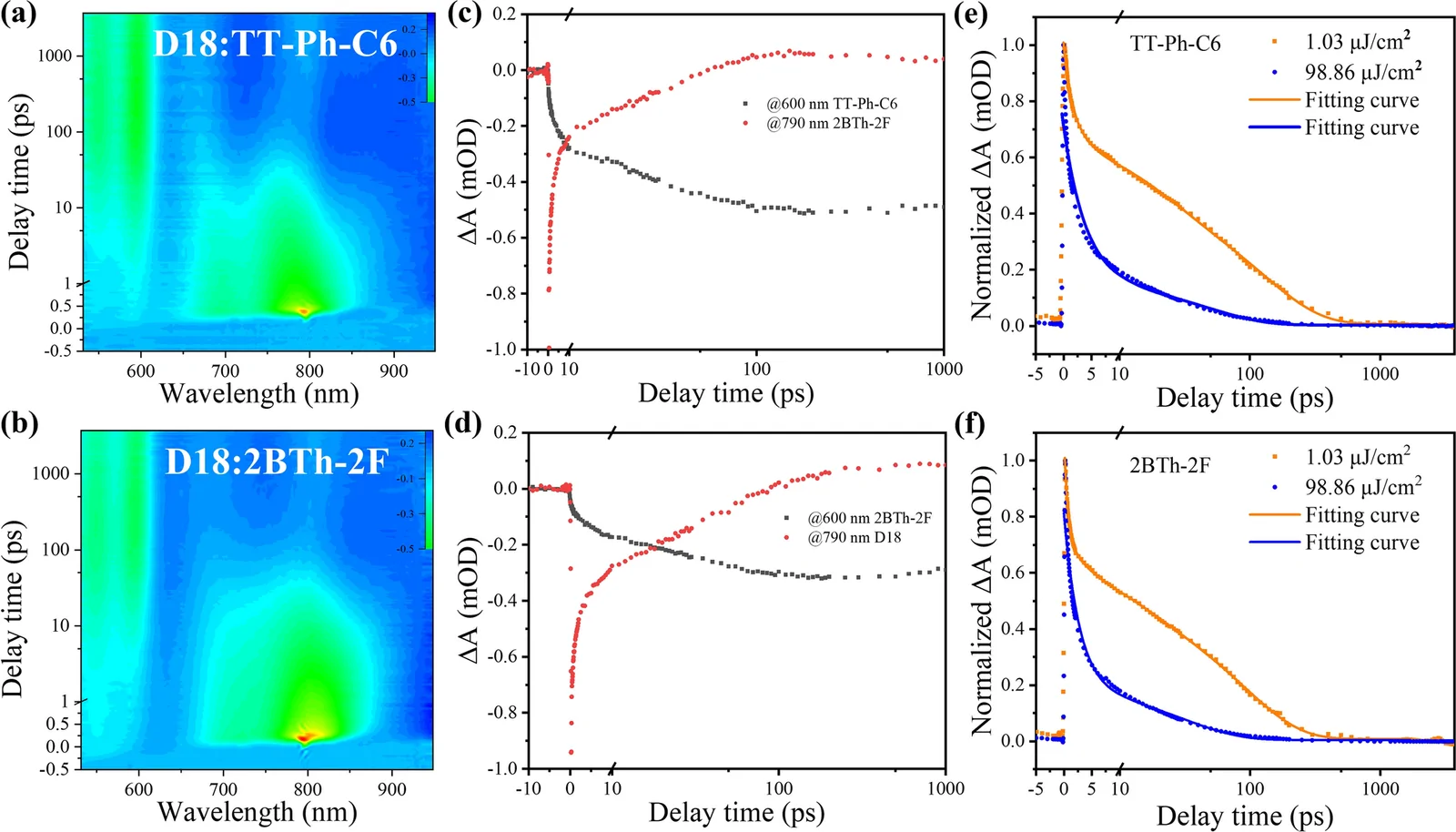
TA image and the corresponding TA spectra of **a** and **c** D18:**TT-Ph-C6**, **b** and **d** D18:2BTh-2F blend films with various decay times; singlet exciton decay dynamics as a function of different excitation fluences for neat films of **e TT-Ph-C6** and** f** 2BTh-2F, measured using time-resolved photoluminescence

To further elucidate the high-efficiency photoelectric conversion mechanism in OSCs employing asymmetric NFREAs, we employed femtosecond time-resolved absorption spectroscopy (fs-TA) to investigate the exciton dynamics within donor–acceptor blend films [20, 48–50], with the results shown in Fig. 4. In Fig. 4a, b, by exciting the acceptor material with a specific 800-nm wavelength light, we were able to elucidate the hole transfer mechanisms in these blend films. Initially, we detected two ground state bleaching (GSB) negative signals for the acceptor molecules, positioned within the 650–750 nm and 750–850 nm spectral ranges, respectively. Subsequently, for the donor D18, efficient hole transfer from the acceptor to the donor resulted in the emergence of two prominent GSB negative peaks at approximately 550 and 600 nm. Furthermore, the positive peak observed around 750–760 nm can be attributed to the absorption characteristics of the charge-separated state, aligning with the data presented in Fig. S4. To gain deeper insights into the dynamic process of hole transfer at the donor/acceptor (D/A) interface, we conducted a fitting analysis of the GSB data at 600 nm using a double-exponential function. The results are displayed in Fig. 4c, d, and detailed fitting parameters are provided in Table S2. Specifically, the *τ*_1_ parameter derived from the double-exponential function fitting signifies the exciton dissociation process at the D/A interface, while the *τ*_2_ parameter reflects the exciton diffusion from the domain to the D/A interface. For a comprehensive understanding of the determined time constants (*τ*) in the blend films, refer to Table S2. In particular, the D18: **TT-Ph-C6** blend film exhibits time constants of *τ*_1_ = 1.40 ± 0.19 ps and *τ*_2_ = 26.91 ± 1.70 ps, whereas the D18:2BTh-2F blend film demonstrates time constants of *τ*_1_ = 2.23 ± 0.27 ps and *τ*_2_ = 35.12 ± 2.87 ps. Moreover, we comprehensively considered the hole transport dynamics within the blend films and the exciton decay dynamics of the pure acceptor, allowing for a qualitative estimation of the hole transfer efficiency (HTE) [51], the detailed calculation process and results are provided in the Table S3, and the hole transfer efficiency increased from 86.3% (D18:2BTh-2F) to 91.2% (D18: **TT-Ph-C6**). Notably, the D18:**TT-Ph-C6** blend film showcases a faster electron transfer rate, which is highly advantageous for charge generation and collection in OSCs, resulting in a significant enhancement in the overall device performance, particularly in terms of the *J*_sc_ and FF.

Subsequently, we utilized fs-TA spectroscopy to precisely measure the exciton diffusion lengths in **TT-Ph-C6** and 2BTh-2F. This sophisticated methodology hinges on the observation of two pivotal physical phenomena: firstly, the decay of monomolecular excitons to the ground state at low excitation densities, and secondly, the bimolecular exciton-exciton annihilation occurring at elevated excitation densities [27, 52]. It is important to highlight that the extent of photoinduced absorption is markedly influenced by the luminescence intensity. When excited by light at a wavelength of 750 nm, the **TT-Ph-C6** and 2BTh-2F films displayed broad-spectrum GSB features, as depicted in Fig. S5. As depicted in Fig. 4e, f, at a lower excitation density of 1.03 μJ cm^−2^, the half-lives of pristine **TT-Ph-C6** and 2BTh-2F films were measured to be approximately 20.17 and 13.30 ps, respectively. However, upon increasing the excitation density to 98.86 μJ cm^−2^, a significant reduction in the half-lives of these films was observed, dropping to 1.31 and 1.61 ps, respectively. Detailed data pertaining to these measurements are provided in Table S4. By employing the formula *L*_D_ = (*Dτ*)^1/2^, we determined the exciton diffusion lengths (*L*_D_) for **TT-Ph-C6** and 2BTh-2F to be 17.17 and 13.36 nm, respectively. The augmentation in exciton diffusion length suggests that charge recombination losses are effectively mitigated in **TT-Ph-C6**-based devices, thereby contributing to an enhancement in *J*_sc_.

### Crystallinity and Morphological Properties

**Fig. 5:**
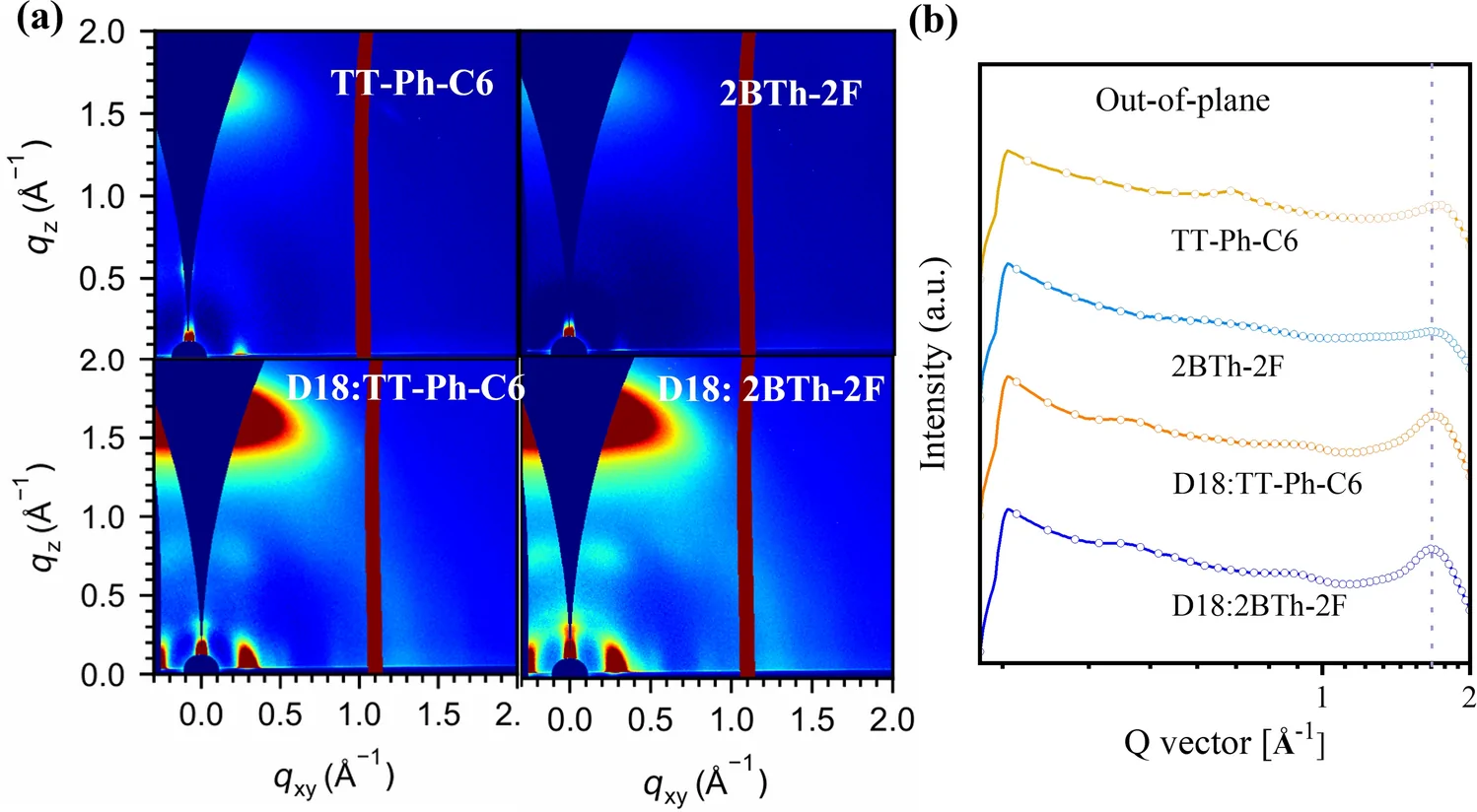
**a** 2D GIWAXS patterns and **b** 1D scattering profiles of the neat **TT-Ph-C6** and 2BTh-2F films and the corresponding blend films

We utilized grazing-incidence wide-angle X-ray scattering (GIWAXS) technique to delve into the crystalline properties, molecular orientation, and intricate molecular packing structures of pure acceptor films and donor–acceptor blend films. The observations unveiled a pronounced face-on molecular orientation, as depicted in Fig. 5. Strikingly, in contrast to the 2BTh-2F film, the pure **TT-Ph-C6** film demonstrated a more pronounced diffraction peak in the out-of-plane (OOP) direction, notably the sharp (010) diffraction peak, indicative of **TT-Ph-C6**’s higher crystallinity. Both the pristine **TT-Ph-C6** and 2BTh-2F films exhibited a prevalent face-on orientation, with (010) diffraction peaks positioned at 1.71 and 1.69 Å^−1^ in the OOP direction, corresponding to *π*–*π* stacking distances of 3.67 and 3.71 Å, respectively. Regarding blend films, the one-dimensional profiles and two-dimensional patterns revealed that both D18:**TT-Ph-C6** and D18:2BTh-2F blend films also displayed face-on molecular orientation, with prominent (010) diffraction peaks at 1.69 and 1.68 Å^−1^ in the OOP direction, corresponding to *π*–*π* stacking distances of 3.71 and 3.73 Å, respectively. Meanwhile, the corresponding one-dimensional map in the in-plane (IP) direction, along with the relevant data, can be found in Fig. S6. To further assess molecular stacking, we employed the Scherrer equation (CCL = 2*π* × 0.89/FWHM, where FWHM denotes the full width at half maximum) to calculate the crystal coherence length (CCL). The results indicated that the (010) diffraction CCL values for **TT-Ph-C6** and 2BTh-2F pure films in the OOP direction were 18.03 and 15.97 Å, respectively, highlighting the superior crystallinity of the **TT-Ph-C6** film. Moreover, the CCL values for the (010) diffraction peaks of D18:**TT-Ph-C6** and D18:2BTh-2F blend films in the OOP direction were 24.30 and 22.35 Å, respectively (Table S5), suggesting that the binary blend film based on **TT-Ph-C6** possessed a more compact and long-range ordered *π*–*π* stacking structure. In conclusion, the asymmetric acceptor **TT-Ph-C6** exhibits higher crystallinity and a more compact *π*–*π* stacking configuration, both of which are conducive to enhancing charge transport efficiency.

The morphology of the thin film plays a pivotal role in determining the performance of the device. Utilizing transmission electron microscopy (TEM) and atomic force microscopy (AFM), we conducted an investigation into the microstructure of the active layer. As depicted in Fig. S7, both methodologies uncovered a uniform phase distribution within the active layer, accompanied by a prominent bicontinuous interpenetrating network structure. The root-mean-square (RMS) roughness values for the D18:**TT-Ph-C6** and D18:2BTh-2F-based blend films were found to be quite comparable, measuring 0.85 nm and 1.05 nm, respectively. Notably, the D18:**TT-Ph-C6** thin film exhibited a tendency to form slightly larger phase separations compared to the D18:2BTh-2F thin film, an observation primarily attributable to the stronger crystallinity of the asymmetric acceptor **TT-Ph-C6**. It is worth mentioning that an appropriate degree of phase separation is conducive to exciton dissociation and transport.

### Photovoltaic Properties of Thick-Film Devices

To further excavate the advantages of the asymmetric acceptor **TT-Ph-C6**, we fabricated thick-film OSCs, and the relevant photovoltaic parameters are listed in Table 3. As Fig. S8 shows, the *V*_oc_ does not change significantly with the thickness of the active layer. However, the *J*_sc_ does vary with film thickness. Specifically, as the active layer thickness increases, **TT-Ph-C6**-based devices display a gradual rise in* J*_sc_ due to improved light absorption. Moreover, the FF values for devices using these acceptors decrease notably as the thickness increases.

**Table 3:** Photovoltaic parameters of devices based on **TT-Ph-C6** and 2BTh-2F with different film thicknesses

Active layer	*V*_OC_ (V)	*J*_SC_ (mA cm^−2^)^a^	FF (%)	PCE (%)	Thickness (nm)
D18:**TT-Ph-C6**	0.87	25.03 (24.09)	69.41	15.18	200
	0.87	25.57 (24.36)	65.35	14.64	300
D18:2BTh-2F	0.89	22.13 (21.89)	62.10	13.20	200
	0.89	22.73 (22.48)	50.90	10.33	300

^a^Integrated from EQE curves

As illustrated in Fig. S8, the EQE spectra of the thick-film devices reveal that the **TT-Ph-C6**-based device outperforms the 2BTh-2F-based one, indicating higher efficiency in light utilization and charge generation for **TT-Ph-C6**. This underscores the significant advantage of **TT-Ph-C6** in maintaining efficient photocurrent generation at greater thicknesses, a crucial factor for enhancing the performance of OSCs. Notably, the asymmetric acceptor **TT-Ph-C6** exhibits a substantially larger exciton diffusion length compared to 2BTh-2F, potentially offering better active layer thickness tolerance and superior photovoltaic performance in thicker active layers. Subsequently, we employed the SCLC method to measure the *μ*_h_ and *μ*_e_ of the thick-film devices. The results, presented in Fig. S9 and summarized in Table S4, indicate that for a 200-nm thick film, the *μ*_h_/*μ*_e_ values for the D18:**TT-Ph-C6** and D18:2BTh-2F blend films are 14.13/14.36 (× 10^−4^ cm^2^ V^−1^ s^−1^) and 9.68/11.19 (× 10^−4^ cm^2^ V^−1^ s^−1^), respectively. When the film thickness is increased to 300 nm, the *μ*_h_ and *μ*_e_ values for the D18:**TT-Ph-C6** blend film rise to 29.85 and 43.83 (× 10^−4^ cm^2^ V^−1^ s^−1^), respectively, while for the D18:2BTh-2F film, they are 19.87 and 32.13 (× 10^−4^ cm^2^ V^−1^ s^−1^), respectively. In the context of thick films, devices based on the D18:**TT-Ph-C6** blend demonstrate enhanced and more balanced hole and electron mobilities, which are vital for achieving high *J*_sc_ and FF.

## Conclusions

We have designed a novel NFREA, **TT-Ph-C6**, featuring asymmetric side chains, which demonstrates substantial improvements in photovoltaic performance compared to the conventional 2BTh-2F. Due to its compact molecular arrangement and enhanced *π*–*π* stacking interactions, **TT-Ph-C6** not only exhibits improved electron mobility but also prolonged exciton diffusion lengths, achieving a remarkable PCE of 18.01% and an FF of 80.10%. This surpasses the performance of devices based on 2BTh-2F with symmetric side chains (16.78%). Impressively, **TT-Ph-C6** exhibits exceptional performance in thick-film configurations. Even at a thickness of 200 nm, it maintains a PCE of 15.18%; when the thickness is increased to 300 nm, the PCE still reaches an impressive 14.64%, marking a new milestone for NFREA materials. In conclusion, this molecular design strategy featuring asymmetric side chains offers a new pathway for developing cost-effective, high-performance NFREAs. It holds significant potential for advancing research and development in OSCs, particularly those that exhibit high efficiency and robustness against thickness variations.

## Supplementary Information

Below is the link to the electronic supplementary material.

Supplementary file1 (DOCX 1501 KB)
