# Supplementary material for: Asymmetric Side-Group Engineering of Nonfused Ring Electron Acceptors for High-Efficiency Thick-Film Organic Solar Cells
Source: Nanomicro Lett. 2025 Nov 10;18:81. doi: 10.1007/s40820-025-01905-y (PMC12597859; doi:10.1007/s40820-025-01905-y)
Supplement: Supplementary file 1 — Supplementary file1 (DOCX 1501 KB) [file 40820_2025_1905_MOESM1_ESM.docx]

Supporting Information

**Asymmetric Side-Groups Engineering of Nonfused Ring Electron Acceptors for High-Efficiency Thick-film Organic Solar Cells**

Dawei Li^1†^, Nan Wei^1†^, Ya-Nan Chen^2^, Xiaodong Wang^2^, Xu Han^4^, Ziqing Bian^1^, Xinyuan Zhang^1^, Zhe Zhang^5^, Wenkai Zhang^4^, Xinjun Xu^1^, Cuihong Li^1^ *, Yahui Liu^2^ *, Hao Lu^3^ *, and Zhishan Bo^1, 2,^ *

^1^ Key Laboratory of Energy Conversion and Storage Materials, College of Chemistry, Beijing Normal University, Beijing 100875, P. R. China

^2^ College of Textiles & Clothing, State Key Laboratory of Bio-fibers and Eco-textiles, Qingdao University, Qingdao 266071, P. R. China

^3^ College of Materials Science and Engineering, Qingdao University, Qingdao 266071, P. R. China

^4^ Department of Physics and Applied Optics Beijing Area Major Laboratory, Beijing Normal University, Beijing 100875, P. R. China

^5^ Laboratory of Eco-environmental Polymer Materials of Gansu Province, College of Chemistry and Chemical Engineering, Northwest Normal University, Lanzhou 730070, P. R. China

^†^Dawei Li and Nan Wei contributed equally to this work.

*Corresponding authors. Email: licuihong@bnu.edu.cn (Cuihong Li); [liuyh@qdu.edu.cn](mailto:liuyh@qdu.edu.cn) (Yahui Liu); [luhao@qdu.edu.cn](mailto:luhao@qdu.edu.cn) (Hao Lu); [zsbo@bnu.edu.cn](mailto:zsbo@bnu.edu.cn) (Zhishan Bo)

**S1 Supplemental Experimental Procedures**

**S1.1 Methods and measurements**

Unless otherwise noted, all chemicals were purchased from Aldrich or Acros and used without further purification. The catalyst precursor Pd(PPh_3_)_4_ was prepared according to the literature and stored in a Schlenk tube under nitrogen atmosphere. Unless otherwise noted, all reactions were performed under an atmosphere of nitrogen and monitored by thin layer chromatography (TLC) on silica gel. Column chromatography was carried out on silica gel (200-300 mesh). ^1^H and ^13^C NMR spectra were recorded on a Bruker AV 400 spectrometer. UV-visible absorption spectra were obtained on a PerkinElmer UV-vis spectrometer model Lambda 750. Elemental analyses were performed on a Flash EA 1112 analyzer. Atomic force microscopy (AFM) measurements were performed under ambient conditions using a Digital Instrument Multimode Nanoscope IIIA operating in the tapping mode. Transmission electron microscopy (TEM) images were obtained with a FEI Technai TF20 (Philip) transmission electron microscopy. The thickness of the blend films was determined by a Dektak 6 M surface profilometer. The electrochemical behavior of the polymers was investigated using cyclic voltammetry (CHI 630A Electrochemical Analyzer) with a standard three-electrode electrochemical cell in a 0.1 M Bu4NPF6 solution in CH_3_CN at room temperature under an atmosphere of nitrogen with a scanning rate of 0.1 V/S. A Pt plate working electrode, a Pt wire counter electrode, and an Ag/AgNO3 (0.01 M in CH3CN) reference electrode were used. The experiments were calibrated with the standard ferrocene/ferrocenium (Fc) redox system and assumption that the energy level of Fc is 4.8 eV below vacuum.

Single crystal structure data were collected on Rigaku XtaLAB P2000 FR-X with a rotating molybdenum anode and a Pilatus 200K detector. Please refer to the attachment document (cif) for details.

Grazing Incidence Wide-Angle X-ray Scattering (GIWAXS) Characterization: the samples for GIWAXS measurements were prepared on silicon substrates and the conditions were the same as the device preparation. GIWAXS experiments were carried out at the beamline 1W1A of Beijing Synchrotron Radiation Facility (BSRF)with an incident beam energy at 8 keV and the beamline BL16B1, BL14B1 and BL02U2 of Shanghai Synchrotron Radiation Facility (SSRF) with an incident beam energy at 10 keV. Scattering data were all collected with a fixed grazing angle of 0.2°. The beam center and sample-to-detector distance were calibrated with LaB6.

**S1.2 Solar Cells Fabrication and Characterization**

OSCs were fabricated with the device configuration of ITO/2PACz/active layer / PDINN/Ag (100 nm). The conductivity of ITO is 20 Ω. A mixture of D18 and acceptor molecule (**TT-Ph-C6**/ 2BTh-2F) in chloroform (CF) was stirred at 100 ^o^C at least 1 hours to ensure sufficient dissolution. On one substrate five cells with an effective area of 0.04 cm^2^ for each were fabricated. Current-voltage characteristics were recorded using an Enli Technology Ltd., Taiwan (SS-F53A) under an AM 1.5G AAA class solar simulator with an intensity of 100 mW cm^-2^ as the white light source and the intensity was calibrated with a standard single crystal Si photovoltaic cell. The temperature while measuring the *J-V* curves was approximately 25 ^o^C. The EQE measurements of OSCs were performed by the solar cell spectral response measurement system QE-R3011 (Enli Technology Ltd., Taiwan), which was calibrated by monocrystalline silicon solar cell in advance.

**S1.3 Fabrication and characterization of the hole/electron-only devices**

Devices with an architecture of ITO/2PACz /active layer/Au was applied to measure the hole mobility. The electron-only devices had a structure of ITO/ZnO/active layer/Al to obtain the electron mobility. The active layers were prepared under the same conditions as the optimal OSCs. According to the Mott-Gurney equation: *J* = 9*εε*_0_*μ*/(8L^3^)V^2^, the mobility can be described as *μ* = 8*JL*^3^/9_0_𝑉^2^. Herein, *ε* denotes the dielectric constant of the blend film based on organic materials, and it is assumed to be constant (3.0); *ε*_0_ = 8.85419×10^-1^2 F m^-1^, which means the permittivity of the vacuum; μ represents the zero-field mobility; *J* is the current density; *L* is the thickness of the film; *V* = *V*_appl_ *V*_bi_, here, *V*_appl_ and *V*_bi_ are the applied voltage to the device and the build-in voltage, respectively.

**S1.4 The correlation between photocurrent density (*J*_ph_) and effective voltage (*V*_eff_)**

By analyzing the correlation between photocurrent density (*J*_ph_) and effective voltage (*V*_eff_). It is evident from the figure that as the *V*_eff_ increases, the *J*_ph_ gradually reaches the saturated photocurrent density (*J*_sat_), signifying that excitons in the device can efficiently dissociate into free charge carriers and be collected by the electrodes. The exciton dissociation probability (*P*_diss_) and charge collection efficiency (*P*_coll_) were calculated using the formula *J*/*J*_sat_, where J is the photoinduced current under short-circuit conditions for *P*_diss_ and the maximum output power for *P*_coll_.

**S1.5 The detailed calculation processes of hole transfer efficiency (HTE)**

The hole transfer in the blend is mainly derived from the ultrafast dissociation of boundary excitons and the diffusion-mediated transfer of domain excitons, which can be expressed as

where and represent the percentages of ultrafast exciton dissociation process and diffusion-mediated hole transfer process, respectively. the ultrafast hole transfer efficiency . While in the domain, only part of the excitons diffuse to the D-A interface for dissociation and hole transfer () and the others return to the ground state () by exciton recombination. Therefore, the efficiency of diffusion-mediated hole transfer process can be expressed as the equation of .

**S1.6 Calculation of exciton diffusion lengths by using modified singlet−singlet exciton annihilation (EEA)**

Femtosecond-transient (fs-TA) absorption data of neat **TT-Ph-C6** and 2BTh-2F films excited with the various excitation intensities was used to calculate diffusion length of the acceptors excitons. In this EEA method, exciton quenchers, which may disturb blend morphology, are not necessary. The model used, assumes that exciton decay via radiative and non-radiative deactivations with intrinsic exciton lifetime constant (*k*) and via bimolecular EEA with a bimolecular decay rate coefficient (*γ*), The thicknesses of **TT-Ph-C6** and 2BTh-2F are 56 and 59 nm, respectively. The relationship is shown as follows:

,

where *n*(*t*), is the exciton density at a decay time of *t*. In order to calculate *k*, a low excitation intensity of 1.03 μJ cm^-2^ was used to ensure that EEA effect was absent in the films. The 2D images and corresponding decay profiles of excitons in **TT-Ph-C6** and 2BTh-2F alloy phases are shown in Figure S5 and Figure 4. Photoexcitation of **TT-Ph-C6** and 2BTh-2F films at 750 nm results in the appearance of a broad ground state bleaching (GSB) signals. As expected, the decay dynamics are approximately monoexponential at low excitation intensities, which obey the following equations:

As shown in Table S4. When at low excitation densities (1.03 μJ cm^-2^), the exciton concentrations decay to *t*_1/2_ of the original values, the corresponding time of **TT-Ph-C6** and 2BTh-2F are 13.3 and 20.17 ps. The corresponding k values are 52.11 × 10^9^ and 34.36 × 10^10^ s^-1^. When a relatively high excitation intensity (98.86 μJ cm^-2^), we can obtain the equations as follows [S1, S2]:

When the excitation power density is 50 µJ cm^-2^, we can that the calculated values of *γ* are 2.03 and 3.91 × 10^-8^ cm^3^ s^-1^ for **TT-Ph-C6** and 2BTh-2F phases. Then, the diffusion coefficient D can be obtained through the following equation:

Where R is the annihilation radius of singlet excitons.

**S1.7 Synthesis Section**

All of the reagents involved were purchased from commercial suppliers and used as received.

Compound 1: In a Schlenk tube that was dry, 3,6-dibromothieno[3,2-b]thiophene (1.00 g, 3.35 mmol), 4-ethylaniline (0.93 g, 7.72 mmol), Pd(OAc)_2_ (38 mg, 0.17 mmol), X-Phos (790 mg, 1.67 mmol), NaOt-Bu (805 mg, 8.40 mmol), and toluene (35 mL) were added. The mixture was stirred at a temperature of 110 °C for 20 hours under a nitrogen atmosphere. After cooling, the mixture was filtered through a short silica gel column in Sand core funnel and the resulting filtrate was dried under reduced pressure. Then, the crude product was purified by flash column chromatography on silica gel column eluting with EA/PE (1:20, v/v) to give compound 1 (1.11 g, 88%). ^1^H NMR (600 MHz, Chloroform-d) δ 7.11 (*d, J* = 8.3 Hz, 2H), 6.98 (*d, J* = 8.4 Hz, 2H), 6.76 (s, 1H), 2.61 – 2.58 (m, 2H), 1.22 (*t, J* = 7.5 Hz, 3H).

Compound 2：A solution of compound 1 (1.00 g, 2.67 mmol) in DMF was prepared and NaH (60% dispersion in mineral oil, 420.90 mg, 10.67 mmol) was added dropwise in the absence of light at 0 °C. After stirring for 5 minutes, raise the temperature to room temperature and stir for 30 minutes. Then add iodine hexane (2.26 g, 10.67 mmol) and stir at 80°C overnight. After quenching with water, DCM extraction and brine washing, the crude product was passed through silica gel column with EA/PE (1:20, v/v) eluent to obtain colorless oil (1.35 g, 95%). ^1^H NMR (600 MHz, Chloroform-d) δ 7.10 (*d, J* = 7.2 Hz, 2H), 6.93 (*d, J* = 7.9 Hz, 2H), 6.45 (s, 1H), 3.65 – 3.60 (m, 2H), 2.63 – 2.58 (m, 2H), 1.85 – 1.78 (m, 2H), 1.66 – 1.62 (m, 2H), 1.58 – 1.52 (m, 2H), 1.40 – 1.37 (m, 2H), 1.20 – 1.17 (m, 3H), 0.89 – 0.87 (m, 3H).

Compound 3：Under conditions of 0°C and in the absence of light, a solution of NBS (716.55 mg, 4.03 mmol) in DMF was added dropwise to a solution of compound 2 (1.00 g, 1.83 mmol) in DMF. The mixture was stirred at room temperature for 16 hours and then poured into water. The organic layer was separated and the aqueous layer was extracted with dichloromethane. The organic layers were collected and dried over anhydrous MgSO_4_. After filtration, the organic solvent was removed under reduced pressure. The crude product was purified by flash column chromatography on silica gel using EA/PE (1:10, v/v) as eluent. This resulted in the formation of compound 3 (1.16 g, yield 90%) as a yellowish solid. ^1^H NMR (600 MHz, DMSO-d6) δ 7.02 (*d, J* = 8.3 Hz, 2H), 6.58 (*d, J* = 8.2 Hz, 2H), 3.70 – 3.53 (m, 2H), 2.53 – 2.48 (m, 2H), 1.67 – 1.43 (m, 4H), 1.29 – 1.26 (m, 4H), 1.12 – 1.09 (m, 3H), 0.83 – 0.79 (m, 3H).

Compound 5：A solution of compound 3 (500 mg, 0.71 mmol), tributyl(6-hexylthieno[3,2-b]thiophen-2-yl)stannane (765.0 mg, 1.50 mmol) in toluene (10 mL) was carefully degassed and recharged with nitrogen before the rapidly addition of Pd(PPh_3_)_4_ (40 mg). The mixture was stirred at reflux over night. After removal of solvent under reduced pressure, the crude product was purified by fast column chromatography eluting with petroleum ether to afford mixed compounds 4 as a yellow oil, which was used directly without further purification.

Then, a solution of compounds 4 in 1,2-dichloroethane (10 ml) was placed under the protection of nitrogen. At 0 ℃, DMF (5 mL) and POCl_3_ (1 mL) was slowly added into the mixture and stirred for 30 minutes. Then heated to 85 ^o^C over night. After cooling to room temperature, saturated sodium bicarbonate solution was added and stirred. The mixture was extracted with dichloromethane for three times; and the combined organic phases were washed with saturated salt solution, dried over anhydrous MgSO_4_, filtered and concentrated under reduced pressure. The residue was chromatographically purified on silica gel column eluting with dichloromethane/petroleum ether (2:1, v/v) to give compound 5 (446.2 mg, 60%) as an orange solid. ^1^H NMR (400 MHz, Chloroform-d) δ 10.02 (s, 1H), 7.34 (s, 1H), 7.12 (*d, J* = 8.6 Hz, 2H), 6.77 (*d, J* = 8.6 Hz, 2H), 3.71 – 3.55 (m, 2H), 3.02 – 2.98 (m, 2H), 2.63 – 2.57 (m, 2H), 1.75 – 1.69 (m, 3H), 1.50 – 1.15 (m, 16H), 0.90 – 0.79 (m, 6H). ^13^C NMR (101 MHz, Chloroform-d) δ 182.21, 145.24, 144.44, 143.78, 141.99, 141.41, 139.05, 135.38, 135.18, 134.87, 133.61, 131.01, 128.94, 128.86, 117.11, 115.12, 65.66, 51.89, 31.66, 31.53, 30.04, 29.08, 28.25, 27.99, 27.92, 27.00, 22.77, 19.28, 15.68, 14.10.

**TT-Ph-C6**: Compound 5 (0.15 g, 0.14 mmol), 2-(5,6-difluoro-3-oxo-2,3-dihydro-1H-inden-1-ylidene)malononitrile (0.10 g, 0.42 mmol), acetic anhydride (0.3 mL), boron trifluoride diethyl etherate (0.2 mL) and dried toluene (20 mL) were added to a Schlenk tube. The mixture was stirred overnight at room temperature. After the reaction was completed, the solvent was removed, and methanol was added for suction filtration. The obtained filter cake was purified by silica gel column chromatography using dichloromethane as the eluent to obtain a black-blue solid compound **TT-Ph-C6** (165 mg, 80%). ^1^H NMR (400 MHz, Chloroform-d) δ 9.00 (s, 1H), 8.53 – 8.49 (m, 1H), 7.66 – 7.62 (m, 1H), 7.38 (s, 1H), 7.15 (*d, J* = 8.6 Hz, 2H), 6.79 (*d, J* = 8.6 Hz, 2H), 3.72 – 3.64 (m, 2H), 3.04 – 3.00 (m, 2H), 2.64 – 2.58 (m, 2H), 1.71 – 1.63 (m, 3H), 1.40 – 1.18 (m, 16H), 0.86 – 0.83 (m, 6H). ^13^C NMR (101 MHz, Chloroform-d) δ 185.98, 159.13, 156.15, 155.66, 152.55, 151.62, 145.55, 143.46, 142.77, 136.70, 136.14, 135.99, 135.53, 135.02, 134.42, 128.99, 120.53, 117.10, 115.41, 115.06, 114.94, 114.63, 68.94, 52.23, 31.65, 31.58, 30.88, 29.94, 29.24, 28.02, 27.99, 27.00, 22.79, 15.68, 14.15, 14.10. MS (MALDI-TOF): m/z Calcd. For C_84_H_78_F_4_N_6_O_2_S_6_: 1470.44 [M], Found 1470.94 [M+].

**S2 Supplementary Figures**

**Fig. S1** Cyclic voltammogram (CV) curves of Fc/Fc+, **TT-Ph-C6** and 2BTh-2F

**Fig. S2** Single crystal diagram of **TT-Ph-C6** at different angles

**Fig. S3** Dependence of *J*_ph_ on *V*_eff_

**Fig. S4** The TA spectra D18:**TT-Ph-C6** and D18:2BTh-2F based film with various decay times

**Fig. S5** The TA images of **TT-Ph-C6** and 2BTh-2F neat film under the excitation of 750 nm, 1.03 and 98.86 μJ cm^-2^

**Fig. S6** The surface morphology of transmission electron microscopy (TEM) and atomic force microscopy (heihgt images) for the D18: **TT-Ph-C6** and D18: 2BTh-2F blend films with 100 nm film thickness

**Fig. S7** Different film thicknesses for J–V curves **a**), EQE curves **b**)

**Fig. S8** Different film thicknesses for the μe transport mobilities (**a, c**) and μh transport mobilities (**b, d**)

**S3 Supplementary Tables**

**Table S1** Hole and electron transport mobilities of **TT-Ph-C6** and 2BTh-2F based devices with 100 nm film thickness

|  | *μ*_h_×10^-4^(cm^2^ V^-1^ s^-1^) | *μ*_e_×10^-4^(cm^2^ V^-1^ s^-1^) | *μ*_h_/*μ*_e_ | Thickness [nm] |
| --- | --- | --- | --- | --- |
| **D18:TT-Ph-C6** | 2.44 | 2.48 | 0.98 | 100 |
| **D18:2BTh-2F** | 1.53 | 1.71 | 0.89 | 100 |

**Table S2** The hole transfer kinetics in blend films (TA)

| Active layer | A_1_ | *τ*_1_ (ps) | A_2_ | *τ*_2_ (ps) |
| --- | --- | --- | --- | --- |
| D18:**TT-Ph-C6** | 0.136 ± 0.01 | 1.4 ± 0.19 | 0.30 ± 0.01 | 26.91 ± 1.70 |
| D18:2BTh-2F | 0.08 ± 0.008 | 2.23 ± 0.27 | 0.18 ± 0.007 | 35.12 ± 2.87 |

**Table S3** The hole transfer efficiency

|  | *c*_1_ | *τ*1（ps） | *c*_2_ | *τ*2（ps） | *η*_1_ | *η*_2_ | *η*_HT_ |
| --- | --- | --- | --- | --- | --- | --- | --- |
| D18: TT-Ph-C6 | 0.403 | 1.4 | 0.597 | 26.9 | 0.99 | 0.85 | 0.91 |
| D18:2BTh-2F | 0.282 | 2.2 | 0.718 | 35.1 | 0.99 | 0.81 | 0.86 |

**Table S4** Detailed parameters of single exciton decay dynamic for neat and the alloy acceptor films

| Materials | | Pump  Energy | |  |  | | k |  |  | *D* | *t* |  |
| --- | --- | --- | --- | --- | --- | --- | --- | --- | --- | --- | --- | --- |
| 2BTh-2F | 1.03 | | 2.80 | | 13.3 | | 52.11 | - | - | - | 220.8 | - |
|  | 98.86 | | 267.72 | | | 1.61 | - | 0.12 | 2.03 | 0.81 | - | 13.36 |
| **TT-Ph-C6** | 1.03 | | 1.90 | | 20.17 | | 34.36 | - | - | - | 189.3 | - |
|  | 98.86 | | 181.93 | | 1.31 | | - | 0.06 | 3.91 | 1.56 | - | 17.17 |

**Table S5** Detailed data of GIWAXS Characterization

| out of plane (010) | | | | |
| --- | --- | --- | --- | --- |
| item | location(Å^-1^) | d-spacing (Å) | FWHM | CCL (Å) |
| **TT-Ph-C6** | 1.71 | 3.67 | 0.31 | 18.03 |
| 2BTh-2F | 1.69 | 3.71 | 0.35 | 15.97 |
| D18:**TT-Ph-C6** | 1.69 | 3.71 | 0.23 | 24.30 |
| D18:2BTh-2F | 1.68 | 3.73 | 0.25 | 22.35 |

**Table S6** Hole and electron transport mobilities of **TT-Ph-C6** and 2BTh-2F based devices with different film thickness

|  | *μ*_h_×10^-4^(cm^2^ V^-1^ s^-1^) | *μ*_e_×10^-4^(cm^2^ V^-1^ s^-1^) | *μ*_h_/*μ*_e_ | Thickness [nm] |
| --- | --- | --- | --- | --- |
| D18:**TT-Ph-C6** | 14.13 | 14.36 | 0.98 | 200 |
| D18:2BTh-2F | 9.68 | 11.19 | 0.86 | 200 |
| D18:**TT-Ph-C6** | 29.85 | 43.83 | 0.68 | 300 |
| D18:2BTh-2F | 19.87 | 32.13 | 0.62 | 300 |

**Table S7** Photovoltaic parameter of **TT-Ph-C6** based devices with different weight ratios

| D18: TT-Ph-C6 | *V*_oc_ (V) | *J*_sc_ (mA cm^-2^) | FF (%) | PCE (%) |
| --- | --- | --- | --- | --- |
| 1:0.8 | 0.87 | 22.87 | 71.60 | 14.26 |
| 1:1 | 0.87 | 23.74 | 73.26 | 15.23 |
| 1:1.2 | 0.88 | 22.73 | 69.39 | 13.89 |

**Table S8** Photovoltaic parameter of **TT-Ph-C6** based devices with different additive concentration of DIB

| Additive concentration  (mg/mL) | *V*_oc_(V) | *J*_sc_ (mA/cm^2^ ) | FF(%) | PCE (%) |
| --- | --- | --- | --- | --- |
| 3.5 | 0.87 | 23.57 | 74.23 | 15.34 |
| 4.0 | 0.87 | 23.75 | 74.82 | 15.54 |
| 4.5 | 0.88 | 23.37 | 74.50 | 15.35 |

**Table S9** Photovoltaic parameter of **TT-Ph-C6** based devices with annealing temperature

| annealing temperature (ºC) | *V*_oc_(V) | *J*_sc_ (mA/cm^2^ ) | FF(%) | PCE (%) |
| --- | --- | --- | --- | --- |
| 80 | 0.88 | 24.21 | 73.34 | 15.71 |
| 100 | 0.88 | 24.75 | 74.66 | 16.34 |
| 120 | 0.88 | 23.78 | 75.27 | 15.89 |

**Table S10** Photovoltaic parameter of **TT-Ph-C6** based devices with different proportions of OX (for CF)

|  | *V*_oc_ (V) | *J*_sc_ (mA cm^-2^) | FF (%) | PCE (%) |
| --- | --- | --- | --- | --- |
| 3% | 0.89 | 24.40 | 76.93 | 16.89 |
| 5% | 0.90 | 24.96 | 80.10 | 18.01 |
| 10% | 0.89 | 23.61 | 77.50 | 16.54 |

**Table S11** Photovoltaic parameters of 10 D18:**TT-PH-C6** devices

|  | *V*_oc_ (V) | *J*_cs_ (mA cm^−2^) | FF (%) | PCE(%) |
| --- | --- | --- | --- | --- |
| 1 | 0.90 | 24.96 | 80.10 | 18.01 |
| 2 | 0.90 | 25.42 | 77.73 | 17.83 |
| 3 | 0.90 | 25.48 | 76.89 | 17.61 |
| 4 | 0.90 | 24.61 | 78.65 | 17.49 |
| 5 | 0.90 | 24.89 | 79.05 | 17.77 |
| 6 | 0.90 | 25.06 | 78.25 | 17.67 |
| 7 | 0.90 | 25.02 | 78.84 | 17.78 |
| 8 | 0.90 | 24.97 | 78.77 | 17.67 |
| 9 | 0.90 | 25.07 | 78.71 | 17.69 |
| 10 | 0.89 | 25.17 | 77.91 | 17.52 |

**Table S12** Third-party certified PCE data of D18:TT-Ph-C6

|  | *V*_oc_ (V) | *J*_cs_ (mA cm^−2^) | FF (%) | PCE (%) |
| --- | --- | --- | --- | --- |
| D18:TT-Ph-C6 | 0.91 | 24.77 | 79.62 | 17.96 |

**Supplementary References**

- H. Cha, S. Wheeler, S. Holliday, S.D. Dimitrov, A. Wadsworth et al., Influence of blend morphology and energetics on charge separation and recombination dynamics in organic solar cells incorporating a nonfullerene acceptor. Adv. Funct. Mater. **28**(3), 1704389 (2018). <https://doi.org/10.1002/adfm.201704389>
- H. Cha, S. Wheeler, S. Holliday et al., Influence of Blend Morphology and Energetics on Charge Separation and Recombination Dynamics in Organic Solar Cells Incorporating a Nonfullerene Acceptor. Adv. Funct. Mater. **28**, 1704389 (2018). <https://doi.org/10.1002/adfm.201704389>
